# Epilepsia Open—September 2023—Announcements

**DOI:** 10.1002/epi4.12803

**Published:** 2023-09-01

## ILAE CONGRESSES

2–6 September 2023

35th International Epilepsy Congress

Dublin, Ireland.

22–26 January 2024

13th ILAE School on Pre‐Surgical Evaluation for Epilepsy and Epilepsy Surgery (EPODES Advanced II).

Brno, Czech Republic.

3–8 March 2024

4th International Training Course on Neuropsychology in Epilepsy

Lyon, France.

7–11 September 2024

15th European Epilepsy Congress

Rome, Italy.

## ILAE WEBINARS

12 September 2023

Webinaire éducatif sur l'épilepsie en français

13 September 2023

Overcoming epilepsy stigma in Africa: Nursing Perspectives

22 September 2023

Epilepsy Educational Webinar

25 September 2023

ILAE Pediatrics Commission Webinar

7 October 2023

ILAE Pediatrics Commission Webinar

9 October 2023

Neuropsychology Webinar

10 October 2023

Webinaire éducatif sur l'épilepsie en français

16 October 2023

SUDEP Task Force Webinar

27 October 2023

Epilepsy Educational Webinar

## OTHER CONGRESSES

9–13 September 2023

11th IBRO World Congress of Neuroscience (IBRO 2023).

Granada, Spain.

13–15 September 2023

Philippine League Against Epilepsy 12th National Epilepsy Congress

Tagaytay City, Philippines.

13–16 September 2023

20th International Congress of Neuropathology

Berlin, German.

17–21 September 2023

8th Global Symposium on Ketogenic Therapies

San Diego, CA, USA.

18–22 September 2023

15th International Epilepsy Colloquium: Ictal Semiology and its Value in Epilepsy Surgery

Cleveland, Ohio, USA.

21–23 September 2023

The Future of Neurophysiology and Epilepsy in Africa

Nairobi, Kenya.

28 September–1 October 2023

23rd WPA World Congress of Psychiatry

Vienna, Austria.

29–30 September 2023

31st Malaysian Society of Neurosciences Annual Scientific Meeting

Kuching, Malaysia.

2–4 October 2023

ILAE British Branch Annual Scientific Meeting Gateshead

Gateshead, United Kingdom.

4–6 October 2023

Genetic epilepsies and other neuronal ion channel disorders: Mechanisms and therapeutic perspectives

Tübingen, Germany & Online.

4–19 October 2023

16th Baltic Sea Summer School on Epilepsy

Online.

8–13 October 2023

10th Eilat Educational Course: Pharmacological treatment of epilepsy

Jerusalem, Israel.

11–13 October 2023

4th International Congress on Mobile Health and Digital Technology in Epilepsy

Lausanne, Switzerland.

13–15 October 2023

2023 CLAE Annual Scientific Meeting

Charlottetown, Prince Edward Island, Canada.

15–17 October 2023

Park City Epilepsy Meeting

Park City, Utah.

25–27 October 2023

Shiraz Annual Epilepsy School (Semiology‐EEG).

Shiraz, Iran.

27–28 October 2023

ASEPA‐ANZAN EEG Teaching Course 2023

Pontianak, Indonesia.

1–3 November 2023

Epilepsy Society of Australia and New Zealand League Against Epilepsy Joint Annual Scientific Meeting

Auckland, New Zealand.

1–3 November 2023

Management of Epilepsy in Resource‐Limited Settings: A Practical Guide and Updates on Current Practice

Online.

9–10 November 2023

1st Iranian School of Basic and Advanced Electroencephalography

Isfahan, Iran.

9–11 November 2023

2nd World Brain Disorders and Neuroscience Summit (BDNS 2023).

Singapore.

10–12 November 2023

ILAE British Branch Comprehensive Update in Refractory Epilepsy (CURE)

Chesham, United Kingdom.

11 November 2023

2023 ILAE British Branch Clinical Epilepsy 1‐Day Course for Doctors in Training

Birmingham, United Kingdom.

17–20 November 2023

Imaging avanzato nello studio dell'epilessia

Bologna, Italy.

25–26 November 2023

ASEPA‐ANZAN EEG Teaching Course

Pakistan with Myanmar Online.

30 November 2023

AES In‐Person Epilepsy Self‐Management Training

Orlando, Florida, USA.

1–5 December 2023

AES Annual Meeting 2023

Orlando, FL, USA.

## 2024

22–26 January 2024

13th ILAE School on Pre‐Surgical Evaluation for Epilepsy and Epilepsy Surgery (EPODES Advanced II).

Brno, Czech Republic.

25–28 February 2024

Alpine EEG Course

Oberstdorf, Germany.

5–8 May 2024

Seventeenth Eilat Conference on New Antiepileptic Drugs and Devices (EILAT XVII).

Madrid, Spain.

12–15 June 2024

62. Jahrestagung der Deutschen Gesellschaft für Epileptologie

Offenburg, Germany.

29 June ‐ 2 July 2024

10th Congress of the European Academy of Neurology

Helsinki, Finland.

